# Penile Paget’s Disease: A Case Report and Review of the Literature

**DOI:** 10.26502/anu.2644-2833025

**Published:** 2020-10-01

**Authors:** RC Flanigan, R Dornbier, ML Quek, M Woods, A Gorbonos, G Gupta, MM Harkenrider, A Solanki, A Badami, E Henry, S Berg, D Bova, GA Barkan, MM Picken

**Affiliations:** 1 The Departments of Urology, Loyola University Medical Center, Maywood, IL, USA; 2 Radiation Oncology, Loyola University Medical Center, Maywood, IL, USA; 3 Medical Oncology, Loyola University Medical Center, Maywood, IL, USA; 4 Radiology, Loyola University Medical Center, Maywood, IL, USA; 5 Pathology, Loyola University Medical Center, Maywood, IL, USA

**Keywords:** Extramammary Paget’s Disease, Adenocarcinoma, Urothelial carcinoma

## Abstract

Extramammary Paget’s Disease (EMPD) is a rare cutaneous, slow growing, intraepithelial adenocarcinoma that can be either primary (intraepithelial arising within the epidermis) or secondary (intraepithelial spread of a visceral carcinoma). Here we present the case of a 63-year-old male with EMPD of the glans penis stemming from underlying urothelial carcinoma. Our treatment decision elected for management with chemotherapy and local treatment with radiation therapy. Subsequent, review of the literature demonstrated a rare disease with a variety of underlying malignancies causing this secondary pathology. Caregivers should be aware of the association of Paget’s disease and urothelial cancer and should have a high index of suspicion that erythematous penile lesions may represent Paget’s disease and that penile biopsies should be performed early in this setting.

## 1. Introduction

Extramammary Paget’s Disease (EMPD) is a rare cutaneous, slow growing, intraepithelial adenocarcinoma that can be either primary (intraepithelial arising within the epidermis) or secondary (intraepithelial spread of a visceral carcinoma) [1]. EMPD has a predilection for apocrine gland-bearing areas including the perineum, vulva, axilla, scrotum, and penis. Only a few cases of penile Paget’s disease have been reported [2]. We describe a case of penile Paget’s disease that arose in a patient with urothelial cancer.

## 2. Case Presentation

A 63-year-old Caucasian man first presented 20 years ago for urologic evaluation of urinary tract infections (UTIs) and nephrolithiasis which were treated with shock wave lithotripsy. In 2011, he was diagnosed with low risk clinically-localized prostate cancer (clinical stage T1cN0M0, initial PSA 4.0 ng/ml, biopsy Gleason score 3+3=6 adenocarcinoma in 1/12 cores) and elected to be managed by active surveillance. Repeat prostatic biopsies a year later showed only atypical small acinar proliferation in 1/12 cores. Saturation prostate biopsy performed in January 2015 was negative for cancer. A CT scan done at the time of his prostate saturation biopsies demonstrated multiple bladder stones for which he underwent cystolithalopaxy and transurethral resection of the prostate (TURP) in February 2015. These procedures documented benign prostatic tissue and a negative bladder barbotage cytology. He continued to have recurrent UTIs. In December 2015, he underwent repeat cystoscopy at which time a papillary growth extending into the prostatic urethra was found. Prostate biopsies at that time again demonstrated benign glands; however, subsequent transurethral resection of bladder tumor (TURBT) demonstrated multifocal non-invasive papillary high-grade Ta carcinoma of the bladder with carcinoma in situ (CIS), deep muscle present but not involved. A CT scan also demonstrated soft tissue infiltration and wall thickening of the right renal pelvis. In January 2016, a restaging TURBT demonstrated residual non-invasive high grade urothelial cancer (deep muscle present but not involved), and right ureteroscopy with biopsies demonstrated high grade urothelial cancer on cytology and brush biopsies, in addition to a renal stone. He then underwent right percutaneous nephrolithotomy, at which time a nephrostomy tube was left in place for antegrade intrapelvic instillation of six induction BCG treatments. In April 2016, repeat right ureteroscopy with biopsy and repeat TURBT showed no residual disease in the right upper tract, but persistent non-invasive high grade urothelial cancer in the bladder. He was counseled as to treatment options and elected to proceed with cystoprostatectomy and urinary diversion.

In June 2016, he underwent radical cystoprostatectomy, bilateral pelvic lymphadenectomy, right ureteroscopy and creation of an orthotopic neobladder, pathology revealing high grade noninvasive urothelial carcinoma with associated carcinoma in situ with negative margins (pathologic stage pTaTisN0Mx). There was no evidence of prostate adenocarcinoma in the final prostatectomy specimen.

**Figure 1:**
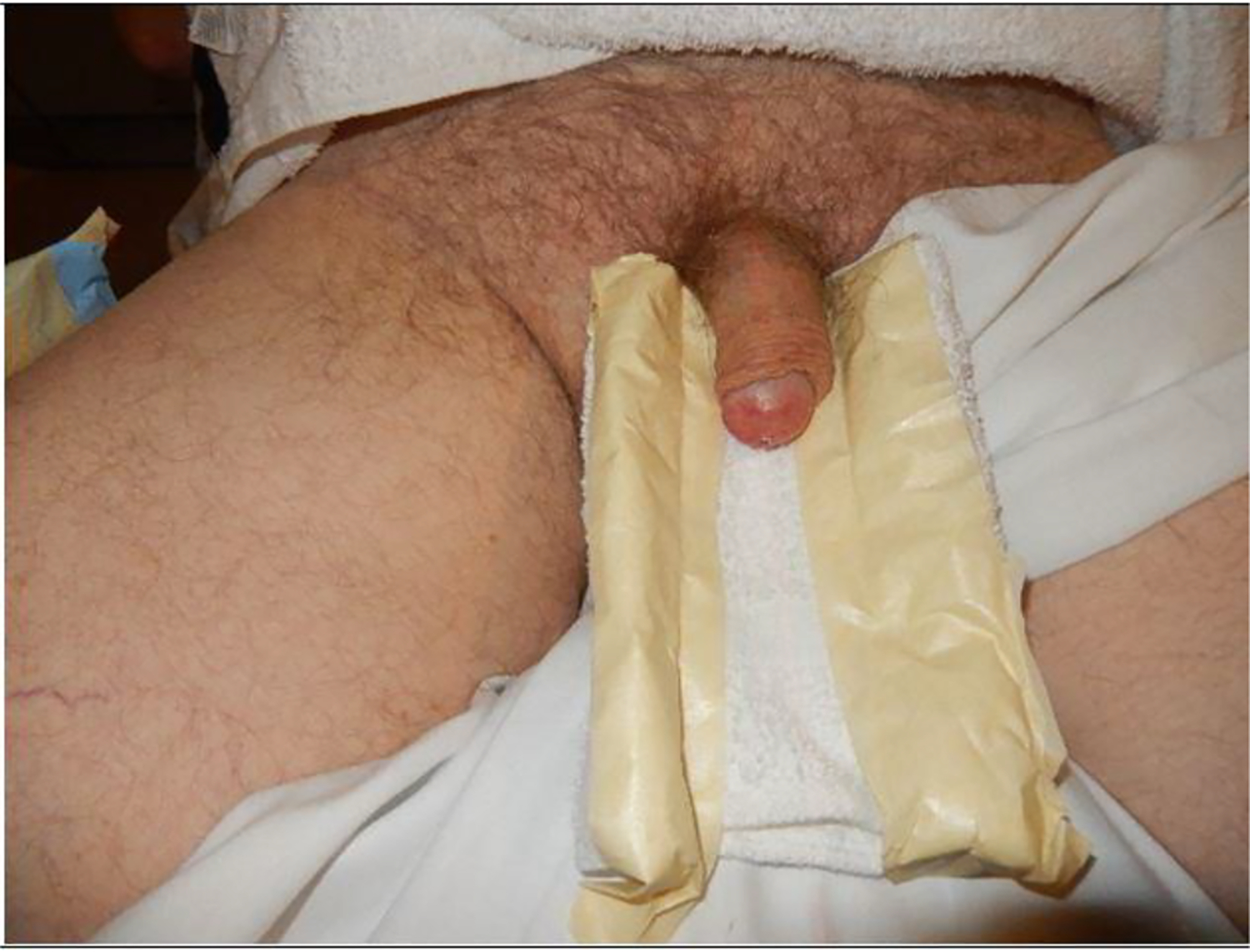
Gross image showing erythema of the penile glans.

In June 2017, he developed bilateral flank pain. A repeat CT scan revealed a left renal pelvic stone with hydronephrosis associated with worsening renal function (Cr 2.3). He underwent left percutaneous nephrolithotomy and the chemical analysis showedthat this was a calcium oxalate and calcium phosphate stone. In December 2019, he presented with a 3 month history of erythema of his distal glans penis that was painless and non-pruritic. (Figure 1).

**Figure 2:**
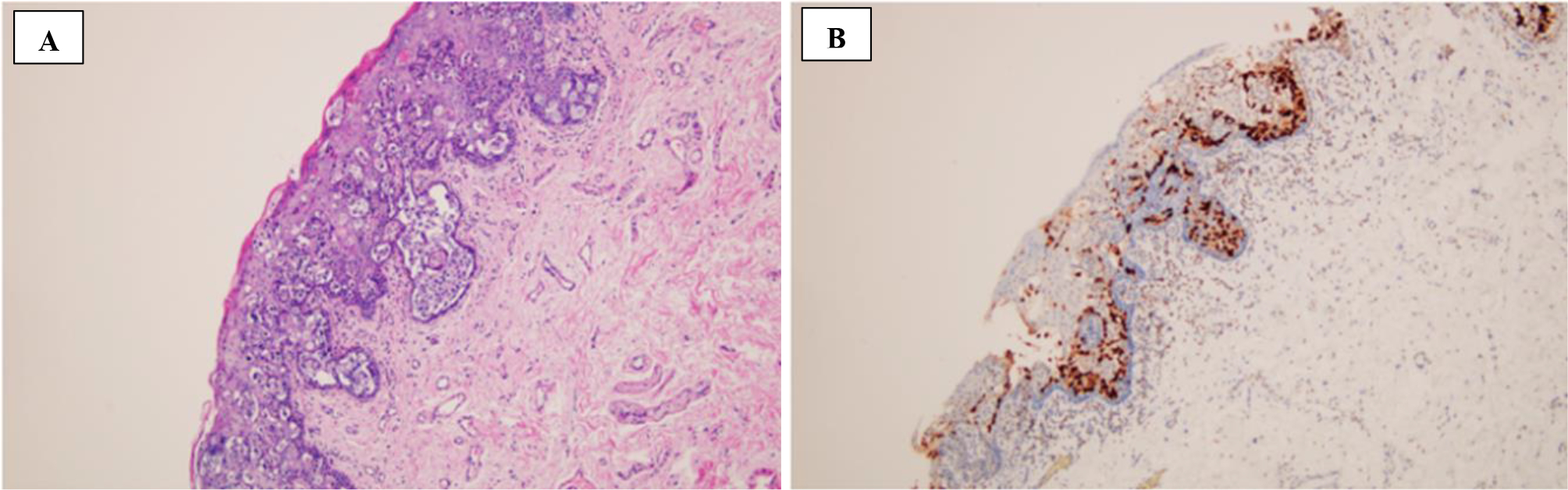
A. H&E stained section showing intraepidermal atypical epithelial cells, some of which with a clear cytoplasm. B. Immunohistochemical stain for GATA3 shows diffuse and strong positivity in the atypical cells. A and B original magnification x100.

This area had been treated with several topical therapies without resolution. He underwent penile biopsies which were positive for Paget’s disease (Figure 2A). Immunostains for CK7, P63, CK20 and GATA3 (Figure 2B) were diffusely and strongly positive, stain for CDX-2 was weaker and less diffuse while stains for HMB-45 and MART-1 were negative. Based on the morphology and the immunophenotype, Paget’s disease secondary to urothelial carcinoma was diagnosed.

A voided urinary cytology was positive for high grade urothelial carcinoma, leading to cystoscopy and urethral biopsies which demonstrated invasive urothelial carcinoma with lymphovascular invasion involving the proximal and mid urethra. FDG PET was negative for nodal or distant metastases and MRI did not show any periurethral invasion of his disease. It was recommended that he undergo combined chemo and radiation therapy which he is receiving currently. He has been treated with 5 FU and MMC in addition to planned 64.8 Gy radiation given in 36 fractions.

## 3. Discussion

Extramammary Paget’s disease (EMPD) of the penis is rare, with only a few previously reported cases. It was first reported in 1889 by Crocker who described a case that affected the penis and scrotum [4]. EMPD is most common in women and in the elderly, with a predilection to apocrine gland-bearing areas. Multiple non-penile sites include the vulva, perianal area, axilla, scrotum, groin, external auditory canal and eyelids. Clinically EMPD presents in a nonspecific way that mimics other forms of dermatitis. The clinical differential diagnosis includes Bowen’s disease, tinea cruris, contact dermatitis, lichen simplex, lichen planus, seborrheic dermatitis and psoriasis. The diagnosis is therefore often delayed. The diagnosis is confirmed histologically, supported by immunohistochemical analysis. The main differential pathologic diagnoses include malignant melanoma, Paget disease, and mycosis fungoides (cutaneous T cell lymphoma). Positive staining for CK7 or CK20 and negative stain for melanoma markers are felt to be the most useful markers [3]. This was also found in our case.

Attempts have been made to classify EMPD based on the origin of the Paget’s cells. Wilkinson and Brown classified vulvar Paget’s disease into two broad groups – primary (of cutaneous origin) and secondary (of non-cutaneous origin) [5]. In an attempt to further help define the extent of disease, prevent unnecessary surgery, and influence the outcome of management, the authors suggest further PD classification as follows:

Primary PD

Type 1 primary intraepithelial without invasion

Type 2 primary intraepithelial with invasion

Type 3 primary intraepithelial as a manifestation of underlying adenocarcinoma of skin appendage origin

Secondary PD associated with underlying non-cutaneous neoplasm

Penoscrotal EMPD has been reported in association with multiple internal malignancies (Table 1). Of the 357 reported cases of penoscrotal EMPD, 76 (21.3%) were found to have an underlying non-cutaneous malignancy, with only 4 cases of urothelial carcinoma reported [6]. EMPD without underlying non-cutaneous malignancy can also metastasize most commonly to lymph nodes, lung and bone.

Our case represents secondary PD that was found in a case of prior and concurrent urothelial cancer. The precise relationship between PD and an underlying malignancy remains uncertain. Reports have suggested that up to 42% of patients harbor a non-cutaneous malignancy [7]. The location of the PD may also relate to the underlying malignancy. For example, penoscrotal and perianal locations are most likely associated with genitourinary and digestive tract cancers [8]. Therefore, following the diagnosis of EMPD, a thorough search for an underlying non-cutaneous malignancy is recommended. EMPD can be treated surgically with wide excision and immediate reconstruction, with radiation therapy and with systemic therapies [6]. After surgery, recurrence rates are high, approximating 60% [9)]. The prognosis is good when the disease is confined to the epidermis but in the presence of dermal invasion it is poor [10]. In our case a decision was made to treat the recurrent underlying urothelial cancer as well as the Paget’s disease bearing area on the distal glans penis with combined chemo-radiation therapy as a penis-preserving therapy.

Radiation therapy was delivered with the approach that the recurrent urothelial cancer in the urethra and the EMPD of likely urothelial origin are related recurrences of his prior multi-focal urothelial carcinoma. As such, doses and treatment volumes were chosen as an extrapolation from our experience with bladder preservation therapy. Therefore, the primary target was defined as the urethra from the orthotopic neobladder to the urethral meatus. The draining lymphatics included the bilateral inguinal, obturator, external iliac, presacral and distal common iliac chains. These targets were treated to a dose of 45 Gy in 25 fractions of 1.8 Gy daily (Mon-Fri) and included the distal 2 cm of the neobladder. The urethra and penile glans were then boosted for an additional 19.8 Gy in 11 fractions which is in line with standard fractionation doses for urothelial bladder cancer in a bladder preservation regimen. All treatments were delivered with intensity modulated radiation therapy (IMRT) in order to maximally spare the organs at risk, specifically the bowel, rectum, proximal neobladder, proximal femurs and bone marrow. Special care was taken to ensure reproducible set up given the mobility of the penis and pelvis. Daily KV imaging ensured alignment of the pelvis and daily cone beam CT to ensured alignment of the penis for each treatment.

The chemotherapy regimen was also selected with the understanding that the urethral disease and EMPD likely represent a recurrence of the previously diagnosed urothelial carcinoma. Therefore, the proposed treatment regimens were extrapolated from those established for definitive bladder-sparing approach for urothelial cell carcinoma of the bladder. Cisplatin-based regimens are generally preferred for the management of urothelial cell carcinomas. When administered as a radio-sensitizer, this can be given as either a single-agent or as a doublet in combination with 5-FU or paclitaxel [9]. Potential toxicities associated with cisplatin include hearing loss, tinnitus, peripheral neuropathy, and renal dysfunction. The degree of toxicity and reversibility can often vary. The combination of 5-FU and mitomycin-C is another commonly used radio-sensitizing regimen for the treatment of bladder cancers and is often preferred for patients who are ineligible for cisplatin-based therapy [10]. The patient in this case reported baseline grade 2 tinnitus and expressed a desire to preserve his current function without exacerbating his pre-existing tinnitus. After an extensive discussion with the patient, the decision was made to proceed with 5-FU and mitomycin-C in combination with radiotherapy for definitive treatment of recurrent urothelial carcinoma.

## 4. Conclusion

We report a case of EMPD of the penis associated with recurrent urothelial cancer. A high index of suspicion is necessary to allow for a timely diagnosis in this rare disease as the clinical picture closely mimics other cutaneous lesions and dermatitis of the penis. In our case, the fact that the penile lesion was refractory to typical cutaneous medications used to treat penile dermatitis led us to perform penile biopsies. Pathology demonstrated Paget’s disease which based on the immunophenotype was suggested to be of urothelial origin. Further study with urinary cytology and biopsies of the urethra in this patient with a history of cystectomy and orthotopic neobladder confirmed the presence of recurrent high grade urothelial cancer of the urethra. Caregivers should be aware of the association of Paget’s disease and urothelial cancer and should have a high index of suspicion that erythemataous penile lesions may represent Paget’s disease and that penile biopsies should be performed early in this setting.

## Figures and Tables

**Table 1:** Location and associated internal malignacy for reports of secondary Extra-Mammary Paget’s Disease.

Reference	Location and # of Patients	Internal Malignancy- type and #*
Chanda [11]	Penoscrotal, 18	PC-2, RCC-1, TCC-1
Yugueros [12]	Penoscrotal, 7	PC-1, RCC-1
Powell [13]	Penis, 1	TCC-1
Zollo [14]	Scrotum, 6	PC-2
Perez [15]	Scrotum, 3	PC-1
Besa [16]	Scrotum, 1	CC-1
Voight [17]	Penis, 2	PC-2
Hayashibara [18]	Penoscrotal, 25	PC-3, BC-3, TC-2, RCC-1, Other-16
Yoon [19]	Penoscrotal, 21	GC-3, CR adenoma-3, BC-1, LC-1
Yang [20]	Penoscrotal, 36	RCC-1
Wang [21]	Penoscrotal, 130	No internal malignancy
Zhang [22]	Penoscortal, 25	No internal malignancy
Li [23]	Scrotum, 1	HCC-1
Zhu [24]	Penoscrotal, 38	CAA-12
Lai [7]	Penoscrotal, 33	CAA-12
Hsu [6]	Penoscrotal, 1	No internal malignancy
Ekwueme [1]	Penis, 1	No internal screening
Inder [25]	Penis, 1	TCC-1
Moretto [26]	Penoscrotal, 6	TCC-1, Rectal-1, PC-1

* BC- bladder cancer; CC-colon cancer; CAA-cutaneous adnexal adenocarcinoma; GC-gastrointestinal cancer; HCC - hepatocellular carcinoma; LC- lung cancer- PC-prostate cancer; RCC-renal cell carcinoma; TCC-transitional cell carcinoma. Hsu et al., Urological Science 24 (2013) 30–33 [6] with additions
